# Arginine-Induced Self-Assembly of Protoporphyrin to Obtain Effective Photocatalysts in Aqueous Media Under Visible Light

**DOI:** 10.3390/molecules24224172

**Published:** 2019-11-18

**Authors:** Mahmood D. Aljabri, Nilesh M. Gosavi, Lathe A. Jones, Pranay P. Morajkar, Duong D. La, Sheshanath V. Bhosale

**Affiliations:** 1 School of Science, RMIT University, GPO Box 2476, Melbourne, Victoria 3001, Australia; s3595670@student.rmit.edu.au; 2 School of Chemical Sciences, Goa University, Taleigao Plateau, Goa 403206, India; nileshgosavi311@gmail.com (N.M.G.); pranay@unigoa.ac.in (P.P.M.); 3 Centre for Advanced Materials and Industrial Chemistry (CAMIC), School of Science, RMIT University, GPO Box 2476, Melbourne, Victoria 3001, Australia; lathe.jones@rmit.edu.au; 4 Institute of Chemistry and Materials, Nghia Do, Cau Giay, Hanoi 100000, Vietnam; duc.duong.la@gmail.com

**Keywords:** protoporphyrin IX, l-/d-arginine, self-assembly, photocatalytic activity, degradation RhB

## Abstract

The fabrication of controlled supramolecular nanostructures via self-assembly of protoporphyrin IX (PPIX) was studied with enantiomerically pure l-arginine and d-arginine, and we have shown that stoichiometry controlled the morphology formed. The nanostructure morphology was mainly influenced by the delicate balance of π-π stacking interactions between PPIX cores, as well as H-bonding between the deprotonated acidic head group of PPIX with the guanidine head group of arginine. PPIX self-assembled with l-/d-arginine to create rose-like nanoflower structures for four equivalents of arginine that were 5–10 μm in length and 1–4 μm diameter. We employed UV-vis, fluorescence spectroscopy, scanning electron microscopy (SEM), X-ray diffraction (XRD), dynamic light scattering (DLS) and Fourier transform infrared spectroscopy (FT-IR) techniques to characterize the resulting self-assembled nanostructures. Furthermore, we investigated the catalytic activity of PPIX and arginine co-assembled materials. The fabricated PPIX–arginine nanostructure showed high enhancement of photocatalytic activity through degradation of rhodamine B (RhB) with a decrease in dye concentration of around 78–80% under simulated visible radiation.

## 1. Introduction

Much of the waste released in industrial effluent pollution is organic, comprising dyes and organics utilized in the leather, plastic, textile and cosmetic industries. According to the World Health Organization (WHO), more than 7 tonnes of synthetic dyes are prepared annually, and most of these dyes are discarded via industrial effluents [1]. In the textile, paper, printing and food product industries, rhodamine B (RhB) is extensively employed [2]. During the industrial manufacturing of RhB, as well as during its utilization in different applications, RhB may be released into the environment on a large scale. Industrial dye effluents are carcinogenic and neurotoxic to animals [3,4], and it has been observed that the natural degradation of such toxic dyes is very difficult and time consuming [5]. To treat dye-polluted industrial effluents, various conventional treatment methods such as adsorption, biological oxidation and coagulation are employed [6,7,8,9,10,11]. However, these dye degradation techniques are time consuming and expensive.

Worldwide researchers have been extensively utilizing heterogeneous photocatalysis for water purification [12,13]. Recently, Wang et al. have employed bare titanium dioxide (TiO_2_) for photocatalysis and compared the results against metal-porphyrin surfactant-assisted self-assemblies with TiO_2_. The nanoflower structure of porphyrin/TiO_2_ displayed a significant improvement, with a rate of photocatalysis almost twice that of TiO_2_ [14]. Porphyrin-based nanostructured materials have been utilized for photocatalysis with a range of various morphologies such as nanosheets, nanoplates, nanorods, nanoflowers and nanobelts [15,16,17,18,19,20,21,22,23].

Our group has reported well-organized porphyrin nanorods of 250-nm lengths via the self-assembly of tetrakis(4-carboxyphenyl)porphyrin (TCPP) on graphene nanoplates using a surfactant-assisted approach [17]. Furthermore, co-assembled nanostructures of TCPP–arginine were utilized for RhB degradation [15]. More recently, we successfully revealed a new design for a free-based porphyrin, 5,10,15,20-tetrakis(pentafluorophenyl)porphyrin (TPFPP), which forms well-ordered crystalline octahedral and rod-like structures through self-assembly using a solvophobic control [16]. The photocatalytic activity of these nanostructures was investigated through the degradation of dyes under visible-light irradiation [16].

Protoporphyrin IX (PPIX) is an iron-free form of hemin, and widely known as a naturally occurring porphyrin. PPIX consists of hydrophobic groups (methyl/vinyl groups) on one side and hydrophilic groups (two propionic residues) on the other [24,25,26]. To date, only a few reports have appeared based on self-assembled nanostructures of PPIX and its derivatives. PPIX molecular building blocks have been assembled in well-ordered shapes such as worm-like structures, nano-spheres, vesicular tubules, nanofibers and micellar fiber structures [27,28,29]. The supramolecular self-assembly of PPIX-based materials is currently gaining substantial interest, and is applicable in a wide range of fields such as chirality induction, charge transfer processes, energy storage, hydrogen generation, molecular recognition, bioimaging and sensing [30,31,32,33,34,35,36,37,38,39,40,41]. However, self-assembled PPIX nanostructures are rarely employed in the photocatalytic degradation of dyes [42].

**Figure 1:**
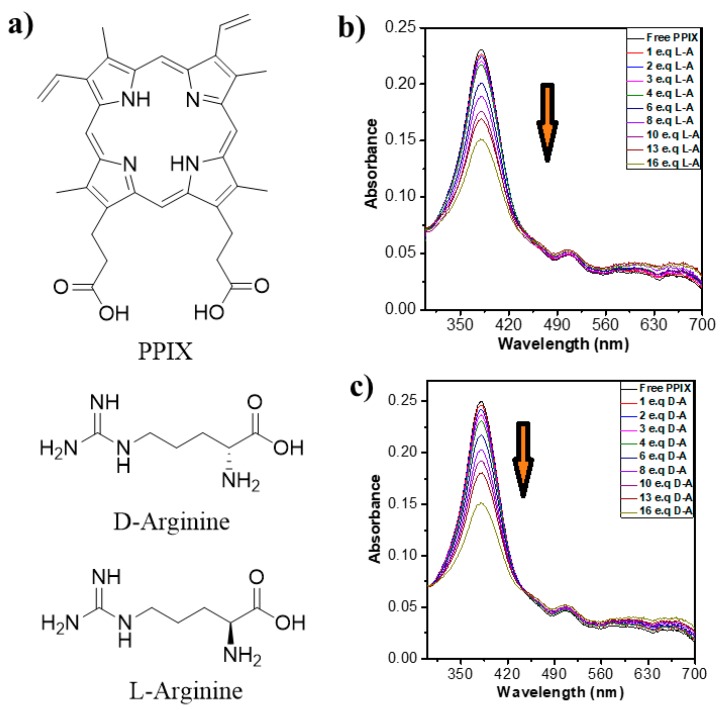
(**a**) Molecular structures of protoporphyrin IX (PPIX) and the d/l-arginine used in this study. Changes in UV-vis absorption spectra of PPIX (pH = 9) upon addition of (0–16 equiv.) l-arginine (**b**) and d-arginine (**c**).

In this manuscript, we report the fabrication of new nanostructures via co-assembly of PPIX and l-/d-arginine (Figure 1a) and their use in dye degradation. PPIX in the presence of l-/d-arginine resulted in well-organized flower-like assemblies. The resulting nanostructures were utilized as catalysts in the presence of simulated visible light to demonstrate the degradation of RhB, and were shown to be efficient. With the addition of further l-arginine (16 equiv.) the absorbance intensity in the UV-vis spectrum of PPIX decreased sharply. Further increases in l-arginine concentration did not lead to any more absorbance changes, indicating that a saturation point was reached. A similar trend in UV-vis absorption changes was observed for PPIX upon addition of d-arginine (Figure 1c). This decrease in the absorbance peak was accompanied by a slight blue-shift in the wavelength maxima of about 4 nm, which may be attributed to H-type aggregation.

## 2. Results

In this work we have employed PPIX in presence of l-/d-arginine for fabricating nanostructures. The self-assembly formation was investigated thoroughly using UV-vis, fluorescence, SEM, DLS, XRD and FT-IR techniques.

### 2.1. Photophysical Properties

#### 2.1.1. UV-Vis Absorption Properties

UV-vis absorbance studies of PPIX in Milli-Q-water under basic conditions at pH ~ 9 and in the presence of the l-/d-arginine solutions in Milli-Q-water with normal pH were performed. The absorbance spectrum of PPIX showed an intense Soret band at 382 nm along with four Q-bands at 500, 550, 600 and 652 nm (Figure 1b,c) [24]. In water, PPIX exhibited a Soret band at 350 nm. However, a significant red-shift was observed upon deprotonation under basic conditions. This indicates that PPIX nanostructures form a face-to-face dimer from the head to the tail of the deprotonated carboxylic acid group periphery [25,26]. The changes in the UV-vis absorption bands of PPIX (3.5 × 10^−5^ M) at different concentrations of l-/d-arginine (0–16 equiv.) are depicted in Figure 1b,c. It can be seen that, at first, a slight decrease occurred in the PPIX Soret band absorbance intensity at 382 nm. With six equivalents of l-arginine, a more significant decrease in absorbance intensity was observed (Figure 1b). A decrease in the absorption bands upon the addition of arginine is ascribed the formation of porphyrin–arginine aggregates leading a decrease of porphyrin concentration in the solution.

#### 2.1.2. Fluorescent Emission Properties

**Figure 2:**
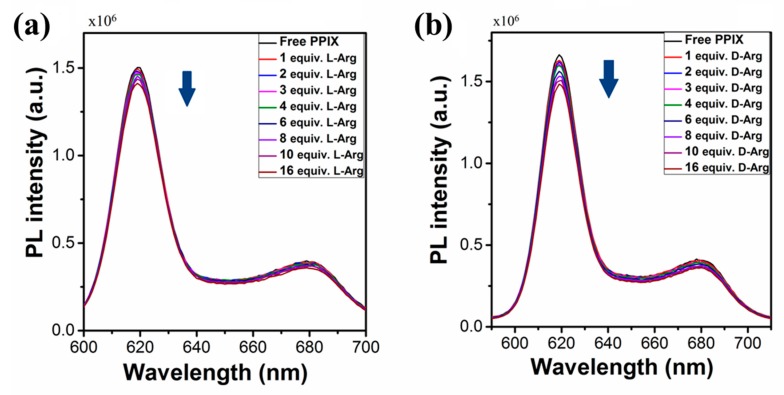
Changes in fluorescence emission spectra of PPIX (pH = 9) upon addition of (**a**) l-arginine and (**b**) d-arginine (0–16 equiv.).

The fluorescent emission properties of PPIX were investigated in water at pH ~ 9 upon excitation at 420 nm. The fluorescence emission spectra of PPIX displays two peaks at 620 and 680 nm (Figure 2a,b). PPIX was tested for fluorescence response with the addition of l-/d-arginine (depicted in Figure 2a,b). Upon addition of 1.0 equiv. of l-arginine to the solution of PPIX, it was observed that PPIX did not display any significant change in the fluorescence emission response (Figure 2a). We collected the fluorescence spectra of PPIX with further additions of l-arginine (2–16 equiv.) and found slight quenching in the photoluminescence (PL) observed as peak intensity changes (Figure 2a). The titration of PPIX solution with d-arginine (1–16 equiv.) showed a similar trend, indicating that the PPIX molecule is involved in a self-assembly process that is accompanied by slight fluorescence quenching.

### 2.2. Scanning Electron Microscopy

**Figure 3:**
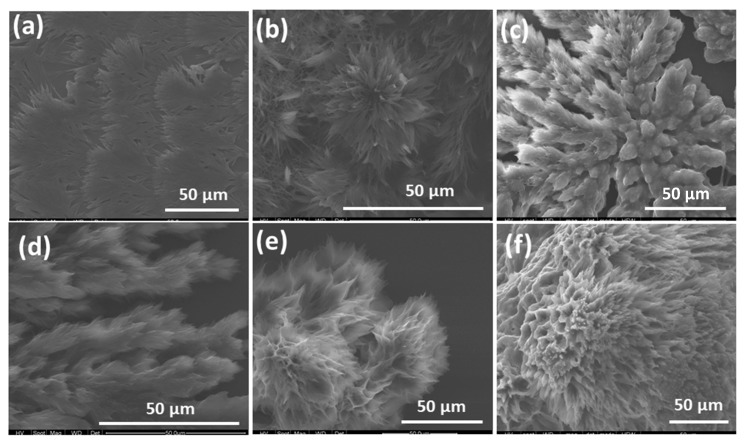
SEM images of self-assembled PPIX with (**a**) 1 equiv. d-arginine, (**b**) 4 equiv. d-arginine, (**c**) 16 equiv. d-arginine, (**d**) 1 equiv. l-arginine, (**e**) 4 equiv. l-arginine and (**f**) 16 equiv. l-arginine.

The supramolecular self-assembled nanostructures formed from PPIX–l-/d-arginine were investigated by scanning electron microscopy (SEM) measurements. The SEM images of self-assembled nanostructures are depicted in Figure 3a–f, Figures S1 and S2 [15]. To systematically investigate the nature of the self-assembly, solutions PPIX–l-/d-arginine were deposited onto silicon wafers followed by solvent evaporation at ambient temperature with ratios of 1:1, 1:4 and 1:16. At a PPIX–d-arginine = 1:1 ratio, flake-like microstructures approximately 10–15 μm in length with a diameter of 1–4 μm (Figure 3a and Figure S1a) were observed. The flake-like microstructures were formed due to the self-assembly of PPIX in a side-by-side fashion, along with both electrostatic interactions and H-bonding between deprotonated carboxylic groups on the peripheral PPIX molecule and positively charged guanidine group on the arginine. On further increasing the amount of d-arginine (4 equiv.), a rose-like nanoflowers morphology of 5–10 μm diameter with nanometer petals was observed (Figure 3b and Figure S1c,d). The growth of nanoflower nanostructures was possibly due to a strong H-bonding interaction between arginine with the –COO^−^ functional group present on the periphery of PPIX, along with π-π stacking on the porphyrin core. A further increase in the molar ratio of PPIX–d-arginine to a 1:16 equiv. led to the formation of the fractal nanostructure shown in Figure 3c. Furthermore, we examined the self-assembly formation between PPIX and l-arginine with 1:1, 1:4 and 1:16 molar ratios. A similar trend of assembly formation was observed as in l-arginine (1 equiv.), with PPIX displaying a fractal-like morphology. Flower-like nanostructure formation took place in the presence of 4 equiv. of l-arginine, with a size several micrometers in diameter. At a PPIX–l-arginine 1:16 ratio, porous nanostructure formation took place, with a size of several micrometers. This is likely attributable to balanced interactions between the H-bonding on one side of the PPIX, and a hydrophobic tail on the other side, accompanied with π-π stacking of the porphyrin core. In comparison with PPIX monomer, which has an amorphous morphology in nature, the self-assembly of PPIX with l-/d-arginine revealed well-defined nanostructures.

### 2.3. X-ray Diffraction Properties

**Figure 4:**
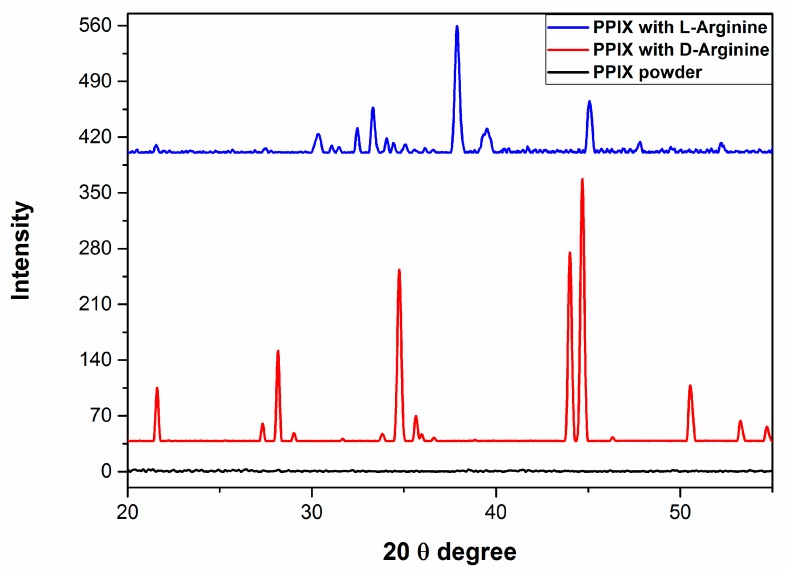
X-ray diffraction patterns of PPIX, PPIX/d-arginine and PPIX/l-arginine at a 1:4 molar ratio.

X-ray diffraction (XRD) was employed to further characterize the crystallinity of the PPIX nanostructure/microstructures after self-assembly with l-/d-arginine. The XRD results are shown in Figure 4. PPIX in its powder form did not exhibit any XRD diffraction. For PPIX–d-arginine (1:4 molar ratio), three significant sharp peaks were observed at 34°, 44° and 45°, along with lower intensity peaks at approximately 22°, 28° and 51°, confirming the crystalline structure of the assembly in the aggregated state. The XRD pattern of PPIX in the presence of 4 equiv. of l-arginine exhibited an intense peak at 38°, with two peaks of lesser intensity at approximately 34° and 46°, indicating the crystalline nature of aggregated nanostructures. The XRD results support the self-assembly formation of PPIX in the presence of l-/d-arginine nanostructures.

### 2.4. Dynamic Light Scattering Measurements

Dynamic light scattering (DLS) was also used in order to confirm the size of the nanostructure material in solution. As shown in Figure S3, PPIX–l-arginine aggregates showed hydrodynamic dimeters of around 900 nm. However, in the presence of d-arginine (4 equiv.), PPIX aggregates displayed a size of around 1200 nm (Figure S3). The DLS results confirm the self-assembly formation observed in SEM images (Figure 3b,e). 

### 2.5. Fourier Transform Infrared Spectroscopy (FT-IR)

**Figure 5:**
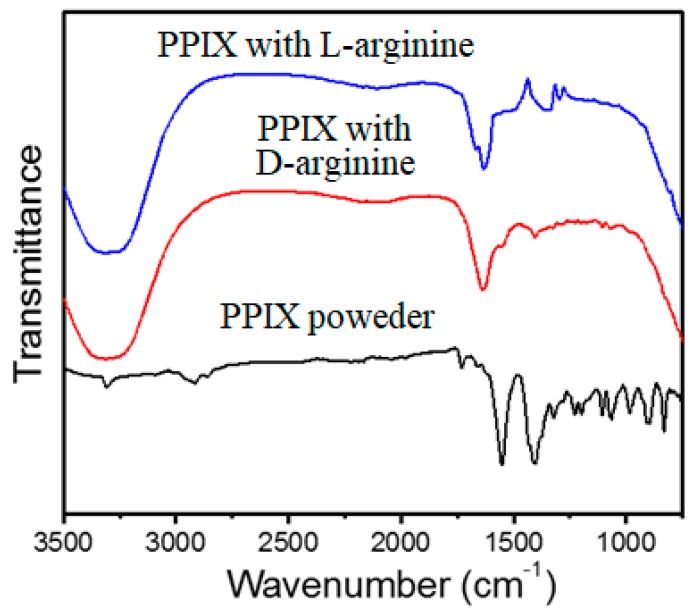
FTIR spectra of PPIX, PPIX/d-arginine and PPIX/l-arginine at a 1:4 molar ratio.

Fourier transform infrared (FTIR) spectroscopy was performed to provide valuable information about the self-assembly formation via molecular level interactions between PPIX and l-/d-arginine. The FTIR spectra of PPIX, and self-assembled PPIX in the presence of l-/d-arginine (4 equiv.) are depicted in Figure 5. PPIX in powder form (black-line) was first characterized by FT-IR and displayed two strong stretching vibration bands at 1410 and 1554 cm^−1^, which can be assigned to C-O-H groups from the carboxylic acid functional group and C=C bonds on the aromatic porphyrin, respectively. There was also one stretching vibration band of C=O from carboxyl groups that appeared at 1732 cm^−1^. The self-assembly of PPIX with l-arginine led to significant decreases in band intensity at 1410 and 1732 cm^−1^, indicating the carboxyl group of PPIX formed H-bonds with arginine (blue line, Figure 5). Furthermore, the stretching vibration of C=C bonds at 1554 cm^−1^ for PPIX shifted to 1638 cm^−1^, which can be attributed to π-π stacking of C=C functional groups in the porphyrin core [26]. The FT-IR spectra of PPIX in the presence of d-arginine were similar to those described above for PPIX in the presence of l-arginine (red solid line) as illustrated in Figure 5, except for the changes in stretching vibration of C=C bonds at 1554 and 1644 cm^−1^, suggesting H-bonding between PPIX and arginine. The FTIR spectra of PPIX at pH 7 are shown in Figure S4, and this figure also clearly shows that the FTIR spectrum of PPIX in water at pH 9.0 was similar to that of the self-assembled PPIX– l-/d-arginine at pH 9.0, respectively; thus, there was no difference in the IR of the deprotonated forms of PPIX at pH 7.0 and 9.0 without arginine and pH 9.0 with arginine, respectively. 

### 2.6. Photocatalytic Performance 

**Figure 6:**
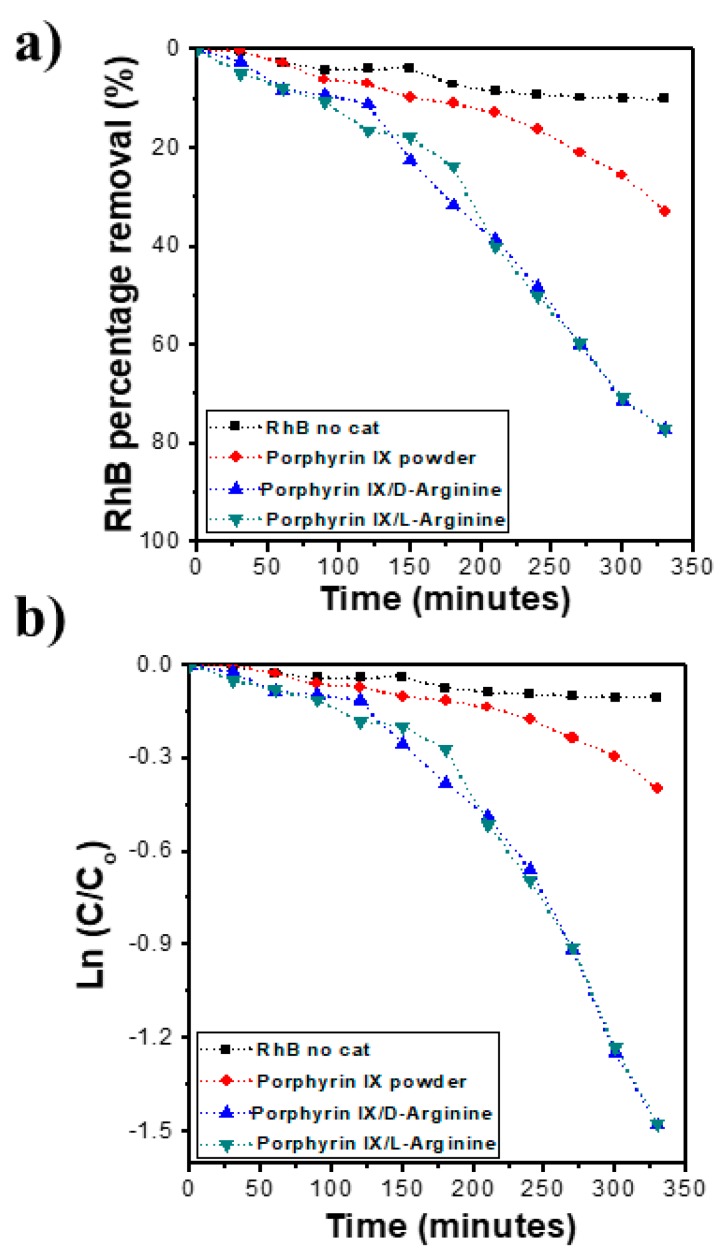
(**a**) Photocatalytic performance of rhodamine B (RhB) degradation and (**b**) the kinetic simulation curves.

The photocatalytic performance of the supramolecular self-assembled nanostructures of PPIX with l-/d-arginine, and their potential application for the destruction of organic pollutants, was examined by the degradation of RhB under the visible-light irradiation. The RhB concentration was determined using UV-vis spectroscopy with PPIX aggregate doses of around 200 mg/L. Self-assembled nanostructures of PPIX l-/d-arginine were directly compared with free PPIX, with results shown in Figure 6. As shown in the Figure 6a, only modest degradation of RhB was observed in the absence of PPIX: 15% degradation after 340 min (dotted black line) in the presence of visible light. In order to examine the RhB degradation of PPIX in absence and presence of l-/d-arginine, similar experiments were performed, and the results revealed that the degradation quantity of the RhB dye increased slightly to around 24% when only PPIX powder (dotted red curve) was utilized. This result implies that minimal photocatalytic activity of the PPIX was found. However, when the self-assembled nanostructure of PPIX with l-/d-arginine (dotted green/dotted blue line) were employed as catalyst for RhB degradation, the concentration of RhB decreased markedly to almost 78% and 80% degradation, respectively, after 340 min of visible-light irradiation (Figure 6a). As expected, upon degradation of RhB, a change in color from pink to clear was observed with naked eye. This confirms the improved photocatalytic performance of the nanostructure obtained from the assembled PPIX in the presence of l-/d-arginine, which was due to the formation of an organic semiconductor via the self-assembly of porphyrin. This resultant organic semiconductor could absorb photon energy from the visible light to generate electron-hole pairs for a photocatalytic reaction.

The photocatalytic degradation of RhB was examined using first order kinetics. The rate of the catalytic reaction was estimated using

Kt = ln Ct/Co (1)

where Kt is the first order reaction; t (min) is the irradiation time of visible light; and Co and Ct are the concentrations of dye initially and after time interval t, respectively.

The kinetic simulation curve of the photocatalytic reaction of RhB was examined to determine the rate constant under different conditions by plotting ln (*C*_t_/*C*_o_) versus time, where C_o_ is the peak intensity at time zero and C_t_ is the peak intensity at time t (Figure 6b). The rate constant of the RhB degradation using the self-assembled nanostructure of PPIX in the presence of l-/d-arginine was 4.49 × 10^−3^ min^−1^.

This improved photocatalytic performance of the nanoflower structures was due to the ordered, high surface-area structure, consisting of aggregated porphyrin cores and induced to assemble via interaction with arginine. Furthermore, the porphyrin core employed in this study was a similar to well-known photoactive molecules in nature (chlorophyll), that have significant photocatalytic activity in many biological systems such as plants and algae [43]. It has been proposed that charge separation of nanostructured assembled materials is enhanced by strong π-π stacking [43].

### 2.7. Photocatalytic Activity Mechanism

**Figure 7:**
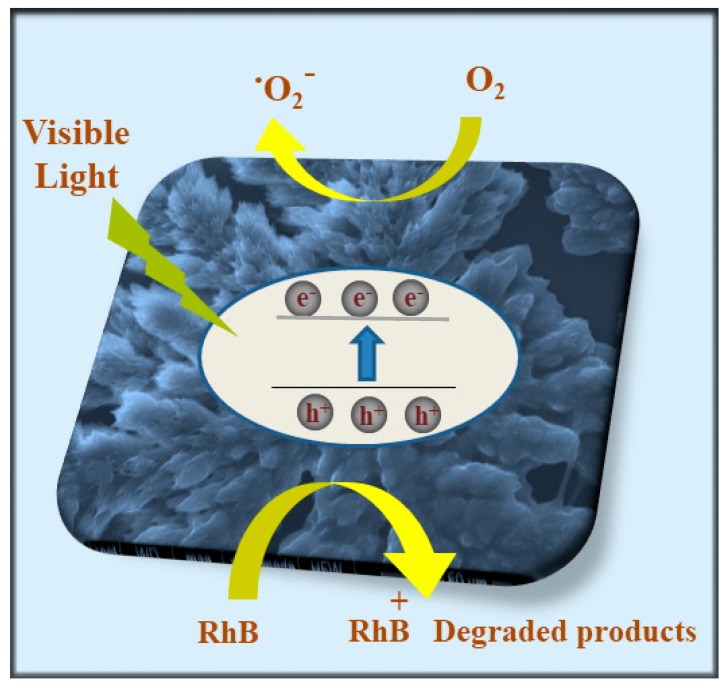
Schematic representation of self-assembled PPIX and l-/d-arginine photocatalyst materials in RhB dye degradation.

The assembly of PPIX, induced via interaction with l-/d-arginine, was successfully undertaken to create a nanostructure through H-bonding between the amino group of arginine with an acid side chain of PPIX. This was followed by π-π stacking of the core to produce nanoflower-like assemblages. This self-assembled superstructure displayed an improved photocatalytic performance compared to the precursor PPIX (as shown in Figure 6). The proposal mechanism of the photocatalytic performance to degraded RhB has been presented and widely discussed earlier by our group [15,16,17,18,19]. Figure 7 illustrates the nanoflower assemblage of PPIX with l-/d-arginine was established, it was irradiated with simulated visible light irradiation and electrons jumped from the valence band (VB) of the PPIX crystals to the conduction band (CB) as a result of the absorption of photo energy in the visible light range. Consequently, pairs of electrons/holes were generated. While the electrons generated from the assemblages of PPIX and l-/d-arginine reduced the oxygen in H_2_O to form ^⋅^O_2_^−^ on the surface of the nanocrystal structure, the generated holes participated in the oxidation of RhB dye to RhB^+^ degraded product. 

## 3. Experimental Section 

### 3.1. Materials and Instruments

All chemicals including protoporphyrin IX and rhodamine B (RhB) were purchased from Sigma–Aldrich (North Ryde BC NSW 1670, Australia) and used as received without further purification.

### 3.2. UV-Vis Absorbance and Fluorescence Spectrometry 

All ultraviolet-visible (UV-vis) and fluorescence measurements of the PPIX with l-/d-arginine were performed in a quartz cell with a 1-cm path length using a Cary 50 Bio spectrophotometer (Agilent Technologies, Santa Clara, CA, United States) and Horiba Jobin Yvon, FluoroMax-4 

Spectrofluorometer (Horiba Scientific, Tokyo, Japan), respectively. The measurements of both UV-vis and fluorescence spectrometry were performed by preparation of 3.5 × 10^−5^ M of PPIX in Milli-Q water with a NaOH solution at pH ~ 9. The guest solutions were prepared in 1.5 × 10^−3^ M of l-/d-arginine in aqueous solution, followed by the addition of various l-/d-arginine separate concentrations from 1 to 16 equiv. into the PPIX solution. The photocatalytic performance of RhB degradation was recorded using UV-vis absorbance measurement. 

### 3.3. Scanning Electron Microscopy

The method used to prepare the scanning electron microscopy (SEM) sample is shown here. PPIX was dissolved in Milli-Q water with NaOH solution at pH ~ 9 with a concentration of 3.5 × 10^−5^ M of PPIX. The other solution was prepared in 1.5 × 10^−3^ M of l-/d-arginine in aqueous solution, followed by addition of various l-/d-arginine separate concentrations from 1 to 16 equiv. into the PPIX solution. After the self-assemblies were formed in the solution, the assemblies were applied drop-wise onto a silicon wafer, allowed to dry at room temperature and coated with platinum before measurement by scanning electron microscopy (SEM) using FEI Quanta 200 SEM (Royal Chemical Institute of Technology University, VIC, Australia) operated at a high voltage (HV) of 30 kV. 

### 3.4. XRD Measurement 

A Bruker AXS D8 Discover instrument (Royal Chemical Institute of Technology University, VIC, Australia) with a general area detector diffraction system (GADDS) using a Cu Kα source was utilized to obtain XRD patterns of PPIX with l-/d-arginine nanoflowers. 

### 3.5. Photocatalytic Investigation 

The photocatalytic performance of PPIX with l-/d-arginine nanoflowers was evaluated by the degradation of RhB in aqueous media. A typical method was employed for the photodegradation experiment by preparing 0.1 mg of co-assembled PPIX and l-/d-arginine in 16 equiv. of aqueous solution. This solution was dispersed in a 20-mL solution of 5 mg L^−1^ RhB dye, while 0.1 mg of PPIX powder was separately dispersed in another 20-mL solution of 5 mg L^−1^ RhB for the purpose of comparison. The resulting dispersions were allowed to be stirred in the dark for 30 min in order to create an adsorption/desorption equilibrium before irradiation. The visible light source for the photocatalytic reaction was a 1500 W air cooled Xenon lamp with a UV cutoff filter (Zolix, Shimogyo-ku Kyoto-shi, Kyoto, Japan), which only allowed wavelengths greater than 400 nm to pass. At the specific time, a 1.5-mL aliquot of the dispersion was taken and centrifuged to remove the photocatalyst. The photocatalytic performance of the resultant aggregation for RhB degradation was evaluated by recording real-time UV-vis adsorption spectra of RhB at a wavelength of 553 nm.

## 4. Conclusions

In summary, we have successfully fabricated high surface area nanostructures of PPIX in the presence of l-/d-arginine via self-assembly to obtain nanoflower-like structures almost 5–10 μm in length and 1–4 μm in diameter. The photocatalytic performance of the nanoflower materials obtained from the co-assembly of PPIX with l-/d-arginine showed an obvious enhanced degradation of RhB under simulated visible light irradiation, and, after 340 min, 78% and 80% decreases in concentration were observed, respectively. The PPIX–arginine nanoflower catalyzed degradation of RhB followed the first-order rate constant value of 4.49 × 10^−3^ min^−1^. This work is a continuation of our previous efforts in this field and shows that the self-assembled materials from PPIX–arginine into ordered structures lead to materials with efficient photocatalytic properties. This work will lead to the development of novel materials via the self-assembly of similar porphyrin structures, and these materials could be applicable in many relevant fields.

## Supplementary Materials

The following are available online: Figure S1. SEM images of PPIX self-assembled with (**a**) 1 equiv. d-arginine, (**b**) 2 equiv. d-arginine and (**c,****d**) 4 equiv. d-arginine; Figure S2. SEM images of self-assembled PPIX with (**a**) 1 equiv. l-arginine, (**b**) 2 equiv. l-arginine and (**c**,**d**) 4 equiv. l-arginine; Figure S3. DLS of PPIX/d-arginine and PPIX/l-arginine in 1:4 molar ratios; Figure S4. pH-dependent FTIR of PPIX with and without arginine.
