# Supplementary material for: Arginine-Induced Self-Assembly of Protoporphyrin to Obtain Effective Photocatalysts in Aqueous Media Under Visible Light
Source: Molecules. 2019 Nov 18;24(22):4172. doi: 10.3390/molecules24224172 (PMC6891641; doi:10.3390/molecules24224172)
Supplement: Supplementary file 1 [file molecules-24-04172-s001.pdf]

Supporting information

Arginine-induced self-assembly of protoporphyrin to obtain effective photocatalysts in aqueous media under visible light

Mahmood D. Aljabri[1], Nilesh M. Gosavi[2], Lathe A. Jones[3], Pranay Morajkar[2], Duong Duc La[4] and Sheshanath V. Bhosale [2,*]

[1] School of Science, RMIT University, GPO Box 2476, Melbourne, Victoria 3001, Australia.

[2] School of Chemical Sciences, Goa University, Taleigao Plateau, Goa 403206, India.

[3] Centre for Advanced Materials and Industrial Chemistry (CAMIC), School of Science, RMIT University, GPO Box 2476, Melbourne, Victoria 3001, Australia.

[4] Institute of Chemistry and Materials, Nghia Do, Cau Giay, Hanoi, Vietnam.

* Correspondence: svbhosale@unigoa.ac.in; Tel +91 (0866) 9609303

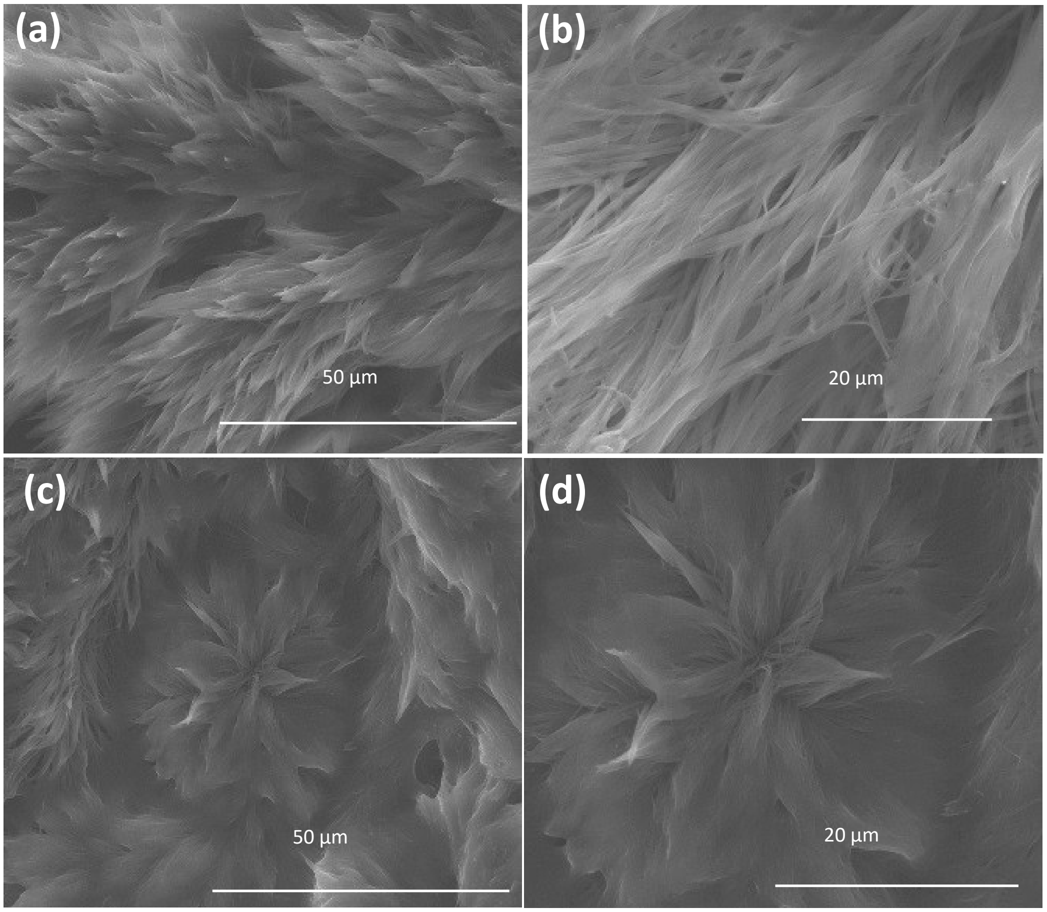

Figure S1. SEM images of PPIX self-assembled with (a) 1 equiv. D-arginine, (b) 2 equiv. D arginine, (c) and (d) 4 equiv. D-arginine.

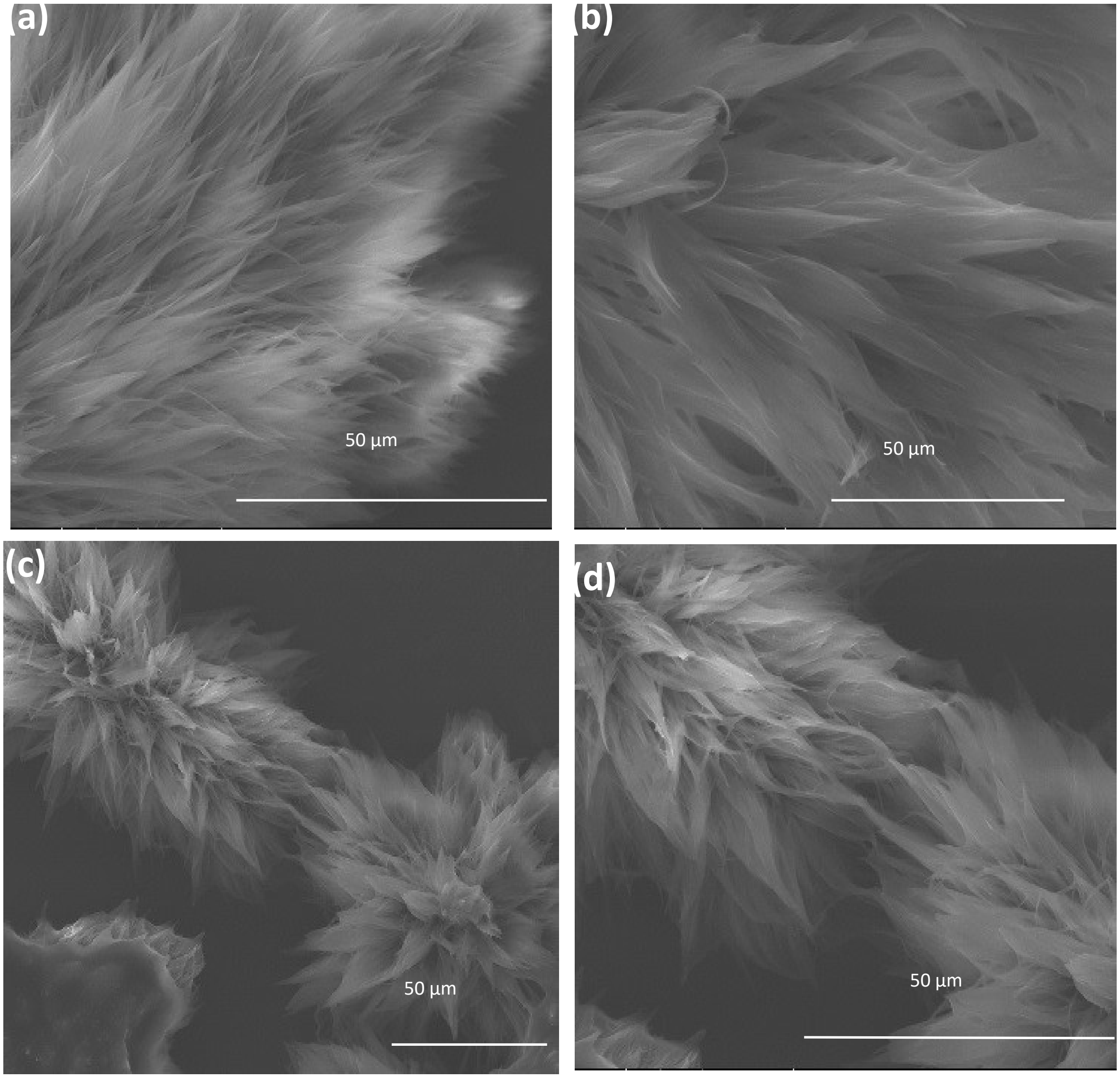

Figure S2. SEM images of PPIX self-assembled with (a) 1 equiv. L-arginine, (b) 2 equiv. L-arginine, (c) and (d) 4 equiv. L-arginine.

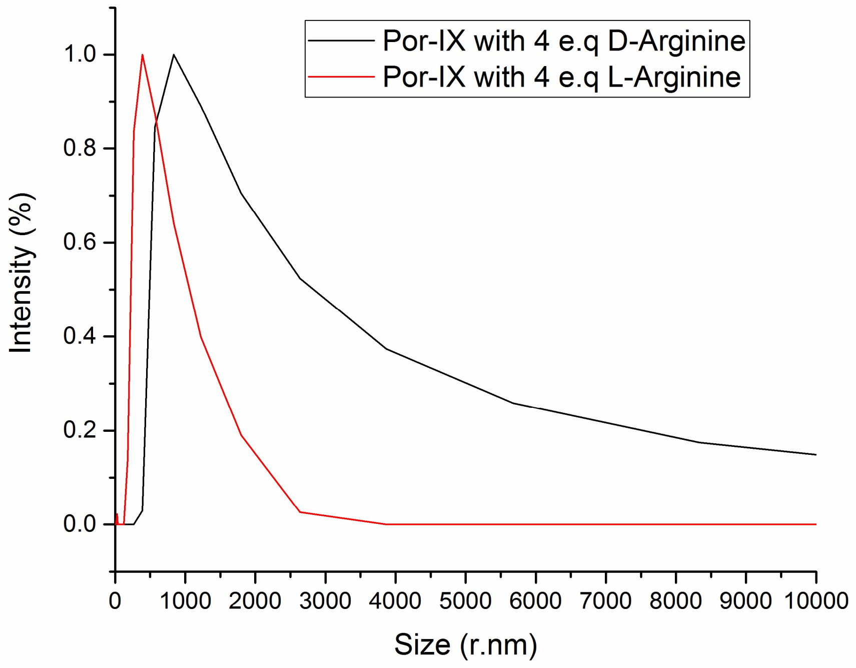

Figure S3. DLS of PPIX/D-arginine and PPIX/L-arginine in 1:4 molar ratio.

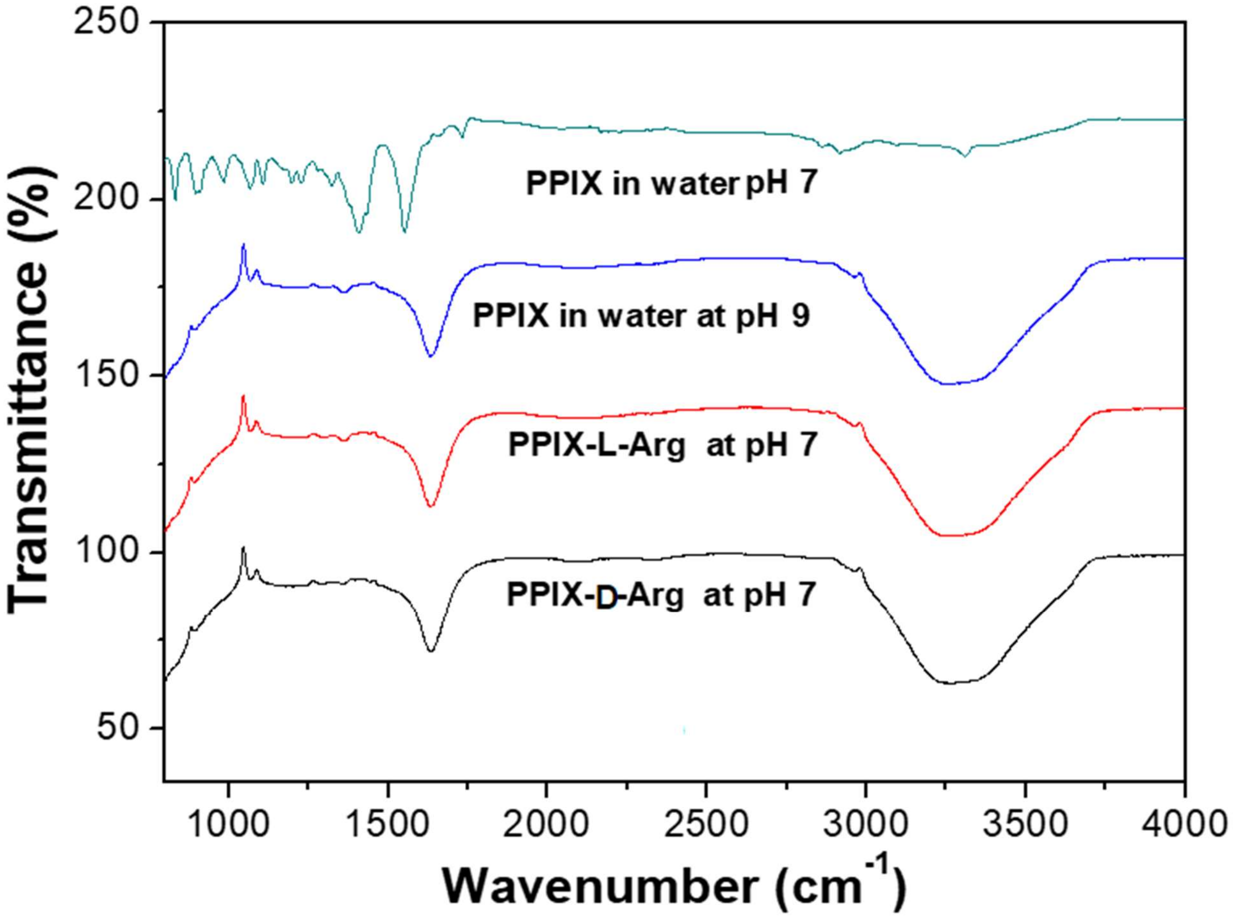

Figure S4. FTIR spectra of PPIX in water at pH 7.0 and 9.0 and self-assembled PPIX with L/D-arginine at 9.0, shows similar behavior.

Reference

S1. La, D.D., Bhosale, S.V., Jones, L.A., Bhosale, S.V. Arginine-induced porphyrin-based self-assembled nanostructures for photocatalytic applications under simulated sunlight irradiation. *Photochem. Photobio. Sci*. **2017**, *16*, 151-154.
